# Effects of α-amylase and coated α-amylase supplementation on growth performance, nutrient digestion, and rumen fermentation in Holstein bulls

**DOI:** 10.3389/fvets.2023.1330616

**Published:** 2024-01-08

**Authors:** Xiaoming Zhang, Feng Xue, Kailin Xu, Qiang Liu, Gang Guo, Wenjie Huo, Yawei Zhang, Cong Wang

**Affiliations:** ^1^College of Animal Science, Shanxi Agricultural University, Jinzhong, China; ^2^DSM Nutritional Products Animal Nutrition & Health, Shanghai, China

**Keywords:** α-amylase, growth performance, nutrient digestion, rumen fermentation, bulls

## Abstract

This study evaluated the impacts of α-amylase (AM) and coated α-amylase (CAM) on bull performance, nutrient digestibility, and ruminal fermentation. This study randomized 60 Holstein bulls of 365 ± 11.5 days of age and 457.5 ± 9.35 kg body weight into three groups: without AM addition, adding AM 0.6 g/kg dry matter (DM), and adding CAM 0.6 g AM/kg DM, separately. This whole experimental period was 80 days, including a 20-day adaptation period and a 60-day data and sample acquisition period. In comparison with the unsupplemented control, dry matter intake (DMI) was unaltered; however, average daily gain (ADG) and feed efficiency (FE) were greater for AM or CAM addition. Bulls receiving AM or CAM supply had greater total-tract nutrient digestibility, ruminal total volatile fatty acids (VFA) content, propionate molar proportion, cellulolytic enzyme and AM activities, and the number of microorganisms. In addition, the activities of AM and trypsin in the jejunum and ileum and glucose, albumin, and total protein concentrations in serum were greater for AM or CAM addition compared to the control. When comparing the supplementation mode of AM, bulls receiving CAM addition had greater ADG and FE. The crude protein and starch digestibility and intestinal AM and trypsin activity were higher, while acid detergent fiber (ADF) digestibility was lower for CAM addition than for AM addition. The lower propionate molar proportion and cellobiase and carboxymethyl cellulase activities, together with *Ruminococcus albus, Ruminococcus flavefaciens*, and *Fibrobacter succinogenes* populations were observed for CAM addition compared with AM addition. However, there were greater glucose, albumin, and total protein concentrations in serum after adding CAM. According to the data, the supply of AM improved ADG, nutrient digestion, and rumen fermentation. Notably, the optimum supplementation mode was in the form of CAM in bulls.

## 1 Introduction

Dietary starch is the main energy source for rumen microorganisms and ruminants. Starch in the diet is degraded to propionate and hydrolyzed to glucose in the rumen and small intestine, respectively (1, 2). Approximately 80% of rumen propionate is used to synthesize glucose by gluconeogenesis (1). Therefore, increasing rumen propionate production and/or intestine starch digestibility can improve dietary energy utilization efficiency and cause an increase in bull performance. It was reported that supplementing α-amylase (AM) in diets increased average daily gain (ADG) in finishing beef (3) and improved lactation performance in dairy cows (4, 5). Moreover, some studies found that AM addition increased rumen butyrate molar proportion (6), stimulated *B. fibrisolvens* D1 growth *in vitro* (7), and increased AM activity and rumen propionate molar proportion of cows (8). Others reported that supplementation with AM increased total-tract digestibility of organic matter (OM), dry matter (DM), neutral detergent fiber (NDF), and crude protein (CP) in cows (4, 9). The synergistic effect of exogenous enzymes and ruminal enzymes increased nutrient degradation (10, 11). These findings showed that supplementing AM in diets could potentially improve nutrient digestibility in the rumen. The improvement was correlated with the stimulating effects of exogenous enzymes on ruminal microbial growth. Nevertheless, no studies evaluated the impact of AM supplements on ruminal fermentation and microflora in bulls. Furthermore, starch digestibility in the small intestine was only 40%–62% in ruminants (12, 13); thus, the exogenous AM supply was required. Studies in weaned pigs and broilers found that exogenous AM addition increased the activities of AM and trypsin in the small intestine (14, 15). However, dietary AM would be destroyed and inactivated in the abomasum (10). Noziere et al. (8) discovered that AM supplementation increased starch degradability in the rumen but did not alter total-tract starch digestion in cows. The coated AM (CAM) supplement can avoid the negative influence of abomasum, releasing 25.3% of AM in the rumen and 69.5% of AM in the intestine. Hence, supplementation with CAM might have a greater increase in ADG than AM addition in bulls.

Based on the research studies above, it is necessary to define the regulatory characteristics of exogenous AM on rumen fermentation and microflora and to find out the proper supplementation mode of AM in ruminant diets. As a result, the study explored the impacts of AM and CAM addition on nutrient digestibility, growth performance, rumen fermentation, microflora, and digestive enzyme activities in the rumen and small intestine of bulls.

## 2 Materials and methods

### 2.1 AM and CAM supplementation

The AM was generated by *Bacillus licheniformis* (Ronozyme RumiStar, DSM Nutritional Products, Basel, Switzerland), and AM activity is 600 KNU/g. 1 KNU is the enzyme level produced during the 2-step α-amylase/α-glucosidase reaction at 37°C and pH 7.0 with 6 μmol p-nitrophenol/min based on 1.86 mM ethylidene-G7-pnitrophenyl-maltoheptaoside (16). The CAM (AM = 40%, hydrogenated fat (C16:0-C18:0 ratio = 2:1) = 37%, calcium stearate = 13%, and bentonite powder = 10%) was produced following Wang et al.’s procedure (17). The release rates of CAM, identified using nylon bag techniques, were 25.3% and 69.5% in the rumen and intestine of ruminal and duodenal fistula bulls, respectively (17).

### 2.2 Animals and experimental design

In this study, our experimental protocols gained approval from the Animal Care and Use Committee of Shanxi Agricultural University. In a randomized block design, 60 Holstein bulls with an age of 365 ± 11.5 days and a body weight (BW) of 457.5 ± 9.35 kg were selected and assigned to three groups: without AM addition, AM 0.6 g/kg DM addition, or CAM 0.6 g AM/kg DM addition, respectively. The AM supplementation amount was determined based on the results of Bachmann et al. (18) and Arturo et al. (19), with the addition of AM 0.5 g/kg DM increasing milk yield in dairy cows. The AM or CAM was added to the premix, mixed with the concentrate, and then incorporated into the total mixed rations (TMR). The basal diet composition and components (Table 1) were prepared as recommended by NASEM (20). Bulls were raised in separate stalls (3 m × 3 m), and fed at 07:00 and 19:00 daily, with free access to water and feed. Additionally, the course of the experiment was 80 days, including a 20-day adaptation period and a 60-day data and sample acquisition period.

**Table 1:** Basal dietary components and nutrient levels (DM basis).

Ingredients	Contents (g/kg DM)
Corn silage	400
Corn grain (ground)	408
Wheat bran	12
Soybean meal	51
Cottonseed meal	45
Distillers dried grains with soluble	24
Corn bran	18
Calcium carbonate	5
Salt	10
Dicalcium phosphate	4
Sodium bicarbonate	18
Mineral and vitamin premix^a^	5
Chemical composition	
Organic matter	945.3
Crude protein	126.8
Ether extract	35.5
Neutral detergent fiber	302.2
Acid detergent fiber	161.0
Starch	432.8
Calcium	7.1
Phosphorus	4.5
Net energy for gain, MJ/kg	5.60

a Included per kg premix: 20,000 mg Fe, 8,000 mg Mn, 7,500 mg Zn, 1,600 mg Cu, 120 mg I, 60 mg Se, 20 mg Co, 820,000 IU vitamin A, 300, 000 IU vitamin D, as well as 10, 000 IU vitamin.

### 2.3 Data collection and sampling procedures

Individual bull BW was determined on days 0/30/60 before feeding at 07:00. We recorded daily DM intake (DMI) for each bull. The feed efficiency (FE) was calculated by dividing ADG by DMI. From days 51 to 57 and for each bull, TMR and refusal samples were gathered daily, and feces (300 g) were gathered from the rectum every 6 h. TMR, refusal, and fecal samples were kept at −20°C, composited by individual animals, dried under 65°C until an unchanged weight was reached, and then ground using a 1-mm filter. The method of AOAC was adopted to measure the contents of DM (method 934.01), OM (method 942.05), acid detergent fiber (ADF; method 973.18), CP (method 990.03), and ether extract (EE; method 920.39) (21). NDF was measured according to the method proposed by Van Soest et al. (22), whereas acid-insoluble ash was identified as depicted by Van-Keulen and Young (23). Starch was enzymatically analyzed in accordance with Hall (24).

On the 58th and 59th days, 200 mL of ruminal fluid was collected from each bull at 04:00, 10:00, 16:00, and 22:00 with the stomach tube. To prevent salivary contamination, we eliminated the initial ruminal fluid (200 mL). An electric pH meter (Sartorius Basic pH Meter PB-10, Sartorius AG) was used to determine the ruminal fluid pH, followed by filtering with the four-layer medical gauze. The collected filtrates were preserved at −20°C and − 80°C, respectively. The AOAC method was adopted to identify ammonia N level (21), while gas chromatography (GC, Trace 1,300; Thermo Fisher Scientific Co., Ltd., Shanghai, China) was conducted to measure VFA using 2-ethylbutyric acid as the endogenous reference.

The ruminal fluid samples preserved at −80°C were employed to determine microbial enzyme activities and populations. We measured the enzyme activities according to the reports of Agarwal et al. (25) and Miller (26). The RBB + C method was used to isolate total microbial DNA from 1.5 mL of ruminal fluid homogenate as Yu and Morrison’s report (27). Extracted DNA purity was determined by agarose gel electrophoresis, while its content was analyzed using a NanoDrop 2000 spectrophotometer (Thermo Scientific, United States). Table 2 presents the primer sequences of microbes. With the use of the regular PCR, DNA standard obtained from samples for PCR assays was acquired by pooling microbial DNA of the treatment set. Subsequently, Pure Link TM Quick Gel Extraction and PCR Purification Combo Kit (Thermo Fisher Scientific Co., Ltd., Shanghai, China) was used for purifying PCR products, while the spectrophotometer was used for quantification. In accordance with the PCR product length as well as mass concentration, we evaluated copy number concentration in every standard substance. The standard curve of the target microorganism was established by 10-fold serial dilutions method (28). The StepOneTM system (Thermo Fisher Scientific Co., Ltd., Shanghai, China) was employed for qPCR amplification and detection. Each sample was measured thrice. The reaction volume of 20 μL was prepared, consisting of SYBR Premix Ex Taq TM II (10 μL, Takara Biotechnology Co., Ltd., Dalian, China), each primer (0.8 μL), template DNA (2 μL), double-standard sterile water (6.0 μL), and ROX Reference Dye II (0.4 μL). RT-PCR conditions were as follows: 2-min initial denaturation under 50°C and 2 min under 95°C, 15 s under 95°C, and 1 min under 60°C for 45 cycles, followed by product elongation. From 60°C to 95°C, the temperature was increased at a rate of 1°C every 30 s.

**Table 2:** List of primers used in RT-PCR assays.

Target species	Sequences of primers (5′)	GenBank accession no.	Size (bp)
Total bacteria	F: CGGCAACGAGCGCAACCC R: CCATTGTAGCACGTGTGTAGCC	AY548787.1	147
Total anaerobic fungi	F: GAGGAAGTAAAAGTCGTAACAAGGTTTC R: CAAATTCACAAAGGGTAGGATGATT	GQ355327.1	120
Total protozoa	F: GCTTTCGWTGGTAGTGTATT R: CTTGCCCTCYAATCGTWCT	HM212038.1	234
*R. albus*	F: CCCTAAAAGCAGTCTTAGTTCG R: CCTCCTTGCGGTTAGAACA	CP002403.1	176
*R. flavefaciens*	F: ATTGTCCCAGTTCAGATTGC R: GGCGTCCTCATTGCTGTTAG	AB849343.1	173
*B. fibrisolvens*	F: ACCGCATAAGCGCACGGA R: CGGGTCCATCTTGTACCGATAAAT	HQ404372.1	65
*F. succinogenes*	F: GTTCGGAATTACTGGGCGTAAA R: CGCCTGCCCCTGAACTATC	AB275512.1	121
*Rb. Amylophilus*	F: CTGGGGAGCTGCCTGAATG R: GCATCTGAATGCGACTGGTTG	MH708240.1	102
*P. ruminicola*	F: GAAAGTCGGATTAATGCTCTATGTTG R: CATCCTATAGCGGTAAACCTTTGG	LT975683.1	74

On day 60 and before the morning feeding, blood samples were collected for all bulls via coccygeal vessels. The samples were subjected to 15-min centrifugation at 2,000*g* and 4°C to obtain serum and were preserved under −20°C. Glucose, total protein, insulin, albumin, urea N, and lactic acid contents were determined using ELISA kits (Shanghai Duma Biological Co., Ltd., Shanghai, China).

On the 61st day, we selected five bulls from each group at random for slaughter. After slaughtering, 50 cm of duodenum, anterior, middle, and posterior segments of the jejunum, and 50 cm of the ileum were quickly harvested. Then, chyme specimens collected from every bull were blended with the equivalent amount of 0.9% NaCl solution and were homogenized for 15 min, prior to 10-min centrifugation (4°C and 11,000×*g*) to obtain supernatants for determining AM, trypsin, and lipase activities by ELISA kits (Shanghai Enzyme Link Biological Co., Ltd., Shanghai, China).

### 2.4 Computation and statistical analysis

The SAS MIXED procedure was used to determine DMI, BW, ADG, and FE (2002; Proc Mixed) (29). The model is shown below:

$$\begin{array}{l} Y_{\mathrm{ijklm}}=\mu +B_{i}+G_{j}+H_{k}+T_{l}+{\left(TG\right)}_{jl}+{\left(TH\right)}_{\mathrm{kl}}+{\left(TGH\right)}_{jkl}\\ \;+R_{m:ijk}+\mathrm{eijklm}\text{.} \end{array}$$

Other measurement results, which were explored, are shown below:

$$Y_{\mathrm{ijklm}}=\mu +B_{i}+G_{j}+H_{k}+R_{m:ijk}+\mathrm{eijkm}\text{.}$$

where *Y_ijklm_* denotes a dependent variable; μ indicates the total average; *B_i_* represents random effect of *i*th block; *G_j_* denotes fixed effects of AM (*j* = 0 or 0.6 g/kg DM); *H_k_* denotes fixed effects of CAM (*k* = 0 or CAM 0.6 g AM/kg DM); *T_l_* denotes fixed effect of feeding time (30 or 60 d); *(TG)_jl_* denotes the interaction effect of feeding time (30 or 60 d) with AM (0 or 0.6 g/kg); *(TH)_kl_* denotes interaction effect of feeding time (30 or 60 d) with CAM (0 or CAM 0.6 g AM/kg); *(TGH)_jkl_* denotes the interaction effect of feeding time (30 or 60 d), AM (0 or 0.6 g/kg), and CAM (0 or CAM 0.6 g AM/kg); *R_m_* denotes random effects of *m*th bull, whereas *εijklm* denotes a residual error. The covariance structure for variables was first-order autoregressive, which was determined by the lowest Akaike’s information criterion (AIC). *p* < 0.05 denotes statistically significant effects.

## 3 Results

### 3.1 Growth performance

0.6 g AM/kg DM supplementation (AM or CAM) did not affect DMI and BW during this trial but elevated ADG (*p* < 0.05) and FE (*p* < 0.05) (Table 3). DMI and BW were similar; however, ADG and FE were greater (*p* < 0.05) after CAM supplementation relative to AM supply.

**Table 3:** Effects of α-amylase (AM) and coated α-amylase (CAM) on DMI, ADG, and FE of Holstein bulls.

	Treatments^a^				*p-*value	
Item	Control	AM	CAM	SEM	Control vs. AM+CAM	AM vs. CAM
DMI (kg/d)						
1–30 d	10.8	11.3	11.3	0.222	0.324	0.165
31–60 d	11.5	12.1	12.6	0.230	0.143	0.125
1–60 d	11.1	11.7	12.1	0.252	0.058	0.258
Body weight (kg)						
1 d	457	458	457	9.61	0.987	0.986
30 d	490	494	499	9.33	0.931	0.925
60 d	526	535	546	9.22	0.715	0.706
ADG (kg/d)						
1–30 d	1.09	1.23	1.40	0.042	0.003	0.004
31–60 d	1.21	1.37	1.56	0.046	0.004	0.006
1–60 d	1.15	1.30	1.48	0.019	0.001	0.001
FE (kg/kg)						
1–30 d	0.101	0.112	0.128	0.004	0.024	0.033
31–60 d	0.104	0.116	0.125	0.004	0.041	0.042
1–60 d	0.103	0.114	0.126	0.003	0.026	0.029

a Control = without AM or CAM addition; AM = AM 0.6 g AM/kg DM; CAM = CAM 0.6 g AM/kg DM.

### 3.2 Total-tract nutrient digestibility and ruminal fermentation

Adding 0.6 g/kg DM AM (AM or CAM) could increase (*p* < 0.05) total-tract digestibility of DM, OM, CP, EE, NDF, ADF, and starch (Table 4). The DM, OM, EE, and NDF digestibility were similar, CP and starch digestibility were greater (*p* < 0.05), while ADF digestibility was lower (*p* = 0.012) after CAM supplementation relative to AM addition.

**Table 4:** Effects of α-amylase (AM) and coated α-amylase (CAM) on nutrient digestion and ruminal fermentation of Holstein bulls.

	Treatments^a^				*p*-value	
Item	Control	AM	CAM	SEM	Control vs. AM+CAM	AM vs. CAM
Nutrient digestibility (%)						
Dry matter	67.6	70.7	72.0	0.669	0.001	0.102
Organic matter	69.9	72.8	73.6	0.602	0.001	0.072
Crude protein	65.4	69.2	72.9	0.348	0.015	0.025
Ether extract	75.2	78.8	80.0	0.350	0.039	0.186
Neutral detergent fiber	56.8	61.4	60.1	0.593	0.011	0.223
Acid detergent fiber	50.6	56.7	54.2	0.717	0.004	0.012
Starch	94.6	95.9	97.3	0.020	0.015	0.009
Ruminal fermentation						
pH	6.61	6.28	6.34	0.044	0.022	0.115
Total VFA (mM)	108	118	114	1.06	0.029	0.092
Mol/100 mol						
Acetate (A)	66.9	66.2	65.8	0.274	0.398	0.385
Propionate (P)	18.5	20.0	19.1	0.198	0.019	0.021
Butyrate	11.5	11.2	10.9	0.230	0.663	0.638
Valerate	1.33	1.38	1.30	0.037	0.683	0.655
Isobutyrate	0.82	0.89	0.81	0.023	0.271	0.255
Isovalerate	1.05	1.21	1.15	0.038	0.277	0.294
A: P^b^	3.62	3.28	3.43	0.042	0.032	0.082
Ammonia N (mg/100 mL)	13.6	9.60	9.88	0.588	0.041	0.458

a Control = without AM or CAM addition; AM = AM 0.6 g AM/kg DM; CAM = CAM 0.6 g AM/kg DM.

b A: P = acetate-to-propionate ratio.

As shown in Table 4, ruminal pH, acetate-to-propionate ratio, and content of ammonia N decreased (*p* < 0.05), whereas the total VFA content and molar proportion of propionate increased (*p* < 0.05). In addition, acetate, butyrate, valerate, isobutyrate, and isovalerate showed unchanged molar proportions for bulls consuming diet supplementation with 0.6 g/kg DM AM (AM or CAM). Bulls receiving CAM addition had similar ruminal pH, total VFA content and acetate, butyrate, valerate, isobutyrate, and isovalerate molar proportions, acetate-to-propionate ratio, and concentration of ammonia N compared with those in the AM group. However, propionate molar proportion was greater (*p* = 0.021) after CAM supplementation compared with AM addition.

### 3.3 Ruminal enzymatic activities and microflora

Adding 0.6 g/kg DM AM (AM or CAM) enhanced the carboxymethyl cellulase, cellobiase, pectinase, and AM activities (*p* < 0.05), but it made no difference to xylanase or protease activity (Table 5). In addition, cellobiase and carboxymethyl cellulase had lower activities (*p* < 0.05), while xylanase, pectinase, AM, and protease had similar activities for the CAM group than for AM addition.

**Table 5:** Effects of α-amylase (AM) and coated α-amylase (CAM) on ruminal microbial enzyme activities and microflora of Holstein bulls.

	Treatments^a^				*p*-value	
Item^b^	Control	AM	CAM	SEM	Control vs. AM+CAM	AM vs. CAM
Microbial enzyme activity^b^						
Carboxymethyl cellulase	0.163	0.182	0.172	0.003	0.013	0.005
Cellobiase	0.358	0.402	0.372	0.007	0.041	0.012
Xylanase	0.469	0.517	0.488	0.010	0.201	0.215
Pectinase	0.723	0.799	0.781	0.012	0.048	0.185
α-Amylase	0.601	0.771	0.745	0.011	0.049	0.112
Protease	1.14	1.21	1.15	0.021	0.142	0.325
Ruminal microflora (copies/mL)						
Total bacteria, ×10^11^	2.05	3.95	3.49	0.223	0.021	0.315
Total anaerobic fungi, ×10^7^	1.77	2.49	2.23	0.091	0.019	0.222
Total protozoa, ×10^5^	1.39	6.51	5.47	0.634	0.032	0.082
*R. albus*, ×10^7^	4.52	6.86	5.61	0.312	0.037	0.032
*R. flavefaciens*, ×10^8^	3.01	4.57	3.58	0.219	0.029	0.028
*F. succinogenes*, ×10^8^	2.66	4.39	3.57	0.231	0.026	0.025
*B. fibrisolvens*, ×10^8^	4.63	7.00	6.85	0.286	0.031	0.135
*P. ruminicola*, ×10^10^	2.13	4.29	4.31	0.277	0.028	0.118
*Rb. amylophilus*, ×10^7^	2.60	3.68	3.29	0.156	0.043	0.062

a Control = without AM or CAM addition; AM = AM 0.6 g AM/kg DM; CAM = CAM 0.6 g AM/kg DM.

b Enzyme activity units are as follows: carboxymethyl cellulase (μmol glucose/min/mL), cellobiase (μmol glucose/min/mL), xylanase (μmol xylose/min/mL), pectinase (μmol D-galacturonic acid/min/mL), α-amylase (μmol maltose/min/mL), and protease (μg hydrolyzed protein/min/mL).

The total bacterial, fungal, protozoa, *Ruminococcus albus*, *Ruminococcus flavefaciens*, *Fibrobacter succinogenes*, *Butyrivibrio fibrisolvens*, *Prevotella ruminicola*, and *Ruminobacter amylophilus* populations were increased (p < 0.05) by 0.6 g/kg DM AM (AM or CAM) supplementation. Bulls receiving AM supplementation had greater (*p* < 0.05) *R. albus*, *R. flavefaciens*, and *F. succinogenes* populations than those receiving CAM addition. Nevertheless, no significant difference was found in total fungal, bacterial, protozoa, *P. ruminicola*, *B. fibrisolvens*, and Rb. amylophilus populations for AM and CAM addition.

### 3.4 Small intestinal enzyme activity

Adding 0.6 g/kg DM AM (AM or CAM) did not affect the activity of lipase in the whole small intestine and the activities of AM and trypsin in the duodenum, but increased (*p* < 0.05) AM and trypsin activities in the proximal, middle, and distal jejunum and ileum (Table 6). The lipase activity in the whole small intestine and AM and trypsin activities in the duodenum were similar, but AM and trypsin activities in the jejunum and ileum increased (*p* < 0.05) after CAM supplementation compared with AM supplementation.

**Table 6:** Effects of α-amylase (AM) and coated α-amylase (CAM) on enzyme activity in the small intestine contents of Holstein bulls (U/g).

	Treatments^a^				*p*-value	
Item	Control	AM	CAM	SEM	Control vs. AM+CAM	AM vs. CAM
Duodenum						
α-Amylase	10.1	10.7	11.3	0.32	0.565	0.536
Trypsin	10.3	11.0	10.7	0.28	0.338	0.365
Lipase	5.21	5.12	5.35	0.16	0.425	0.528
Proximal jejunum						
α-Amylase	11.3	12.1	13.7	0.28	0.012	0.009
Trypsin	12.5	14.0	15.2	0.15	0.015	0.008
Lipase	5.98	6.05	6.11	0.12	0.288	0.328
Middle jejunum						
α-Amylase	13.6	15.9	17.4	0.42	0.025	0.015
Trypsin	18.7	19.6	21.8	0.26	0.011	0.005
Lipase	12.4	12.6	12.6	0.22	0.415	0.436
Distal jejunum						
α-Amylase	12.9	14.3	16.6	0.55	0.019	0.011
Trypsin	17.3	18.5	19.4	0.18	0.009	0.025
Lipase	9.52	9.68	9.63	0.09	0.125	0.153
Ileum						
α-Amylase	10.6	12.8	14.2	0.43	0.012	0.005
Trypsin	11.3	12.5	13.4	0.12	0.006	0.002
Lipase	7.26	7.38	7.33	0.15	0.355	0.288

a Control = without AM or CAM addition; AM = AM 0.6 g AM/kg DM; CAM = CAM 0.6 g AM/kg DM.

### 3.5 Blood parameters

Adding 0.6 g/kg DM AM (AM or CAM) elevated (*p* < 0.05) glucose, albumin, and total protein levels, but it made no difference to insulin, urea N, and lactic acid levels in the blood (Table 7). Bulls receiving CAM supplementation exhibited increased (*p* < 0.05) glucose, albumin, and total protein levels compared with bulls receiving AM; however, no difference was observed in insulin, urea N, and lactic acid levels between the two groups.

**Table 7:** Effects of α-amylase (AM) and coated α-amylase (CAM) on blood metabolites of Holstein bulls.

	Treatments^a^				*p*-value	
Item	Control	AM	CAM	SEM	Control vs. AM+CAM	AM vs. CAM
Glucose (mmol/L)	3.06	3.49	3.95	0.131	0.020	0.012
Insulin (mIU/L)	10.6	11.4	12.2	1.58	0.235	0.215
Total protein (g/L)	67.5	80.1	89.8	3.19	0.030	0.048
Albumin (g/L)	34.7	38.8	42.1	1.10	0.032	0.025
Urea nitrogen (mg/L)	172	148	159	8.52	0.439	0.486
Lactic acid (mg/L)	220	238	243	8.35	0.911	0.882

a Control = without AM or CAM addition; AM = AM 0.6 g AM/kg DM; CAM = CAM 0.6 g AM/kg DM.

## 4 Discussion

The present study explored the impact of supplementing AM in bull diets on performance, nutrient digestibility, intestinal digestive enzyme activities, ruminal fermentation, and blood metabolites. The dietary added fat, resulting from the coated CAM, was 0.55 g/kg DM and had a weak impact on the growth performance, nutrient digestion, and rumen fermentation of bulls. Thus, the effects of added fat from CAM were not discussed.

When AM or CAM was supplemented in bull diets, the response of DMI was limited and consistent with the results in finishing steers (30) or dairy cows (8). As a result, the increase in ADG was caused by the increasing nutrient digestibility as well as rumen total VFA level. Furthermore, such increased total-tract starch digestion, rumen propionate molar proportion, and blood glucose concentration indicated that the addition of AM or CAM improved the energy supply efficiency of starch. The energy utilization efficiency of dietary starch was positively associated with the starch degradation rates in the small intestine and rumen (31). Defrain et al. (32) also found that AM supplementation tended to increase blood glucose concentration in postpartum dairy cows. The current results suggested that the dietary addition of AM or CAM at 360 KUN AM/kg DM improved feed utilization efficiency in bulls as evidenced by the increase in FE. Similarly, other studies reported that 110 or 210 KNU AM/kg DM supplementation increased ADG in finishing steers (3), and 300 KNU AM/kg DM addition increased feed efficiency in dairy cows (9, 19). Nevertheless, DiLorenzo et al. (30) discovered that AM addition at 600 KNU/kg DM made no impact on ADG and FE. Such different findings might be associated with different AM addition levels. Research found that production performance increased quadratically with increasing supplementation levels of AM in steers (3) or lactating Holstein cows (6).

In agreement with the results in dairy cows (4, 8, 9), dietary AM or CAM addition increased the total-tract digestibility of nutrients. Nutrient digestibility response was related to the enhanced ruminal carboxymethyl cellulase, cellobiase, pectinase, and AM activities as well as AM and trypsin activities within the small intestine. According to these findings, AM or CAM supplementation improved nutrient digestibility in the small intestine and rumen. Such enhanced small intestine trypsin activity might be associated with an improvement in starch digestibility with AM or CAM addition as starch had a negative effect on intestinal trypsin activity (33). Similarly, studies in weaned pigs and broilers found that exogenous AM addition increased the activities of AM and trypsin in the small intestine (14, 15).

The reduction of rumen pH was observed with AM or CAM addition. The average value of rumen pH for bulls receiving AM or CAM addition was 6.31, which was suitable for nutrient degradation and microbial growth (34, 35). The decreased pH was related to the increased rumen total VFA level (36). Total VFA levels were changed due to positive responses of rumen enzyme activities and microbial populations, indicating the stimulatory effects of exogenous AM on nutrient degradation and microbial growth. The supplementary AM hydrolyzed starch to maltodextrins, which was used as a substrate for microbial growth (3). Similarly, other studies reported that AM addition tended to increase the total VFA concentration in cows fed high-starch diets (8). While a limited response in acetate molar proportion was observed, the acetate concentration increased to 72.3, 78.1, and 75.0 mM for the control, AM, and CAM groups, respectively. The result conformed to the changes of ADF and NDF apparent digestibility, caused by the increase in microbial populations and cellulolytic enzyme activity. Rumen carboxymethyl cellulase, cellobiase, and pectinase are secreted by fungi, protozoa, *F. succinogenes, B. fibrisolvens, R. flavefaciens,* and *R. albus*, which are used to hydrolyze fiber to acetate (37). The elevated propionate molar proportion, coupled with the reduced acetate-to-propionate ratio, suggested that dietary AM or CAM addition altered the rumen fermentation pattern for further propionate production. These results conformed to the changes in AM activity as well as amylolytic bacterial populations, including *B. fibrisolvens, P. ruminicola*, and *Rb. amylophilus* with AM or CAM addition. Moreover, the increase in AM activity was mainly caused by the positive responses of amylolytic bacteria, showing a synergistic effect of exogenous AM and rumen microbes, as reported by Noziere et al. (8). Similarly, other studies indicated that AM supplementation increased ruminal propionate molar proportion and AM activity in dairy cows (8) and stimulated *B. fibrisolvens* D1 growth *in vitro* (7). However, according to Tricarico et al. (6), AM addition increased the butyrate and acetate molar proportions and the acetate-to-propionate ratio. The inconsistent results may be caused due to the differences in diet composition, especially in starch content.

The decreased rumen ammonia N content did not conform to the unaltered protease activity or the elevated protozoa and protein-degrading bacterial populations (*B. fibrisolvens*, *Rb. amylophilus*, and *P. ruminicola*). Given the positive responses of blood concentrations of albumin and total protein and the limited response of blood urea nitrogen, the reduction of rumen ammonia N content might be caused by an elevation of microbial protein production. Moreover, the elevation of the total VFA level increased the carbon skeleton and energy supply to facilitate microbial protein generation. Furthermore, as found by Gado et al. (38), supplementation of exogenous enzyme mixture including AM increased the duodenal microbial N flow in Brown Swiss.

Bulls fed diets added with CAM had greater ADG and FE compared with those receiving AM supply, indicating that AM should be supplied in the form of CAM, as rumen total VFA concentration was similar for both CAM and AM additions. The greater ADG was correlated with the greater total-tract starch and CP digestibility. In addition, it was caused by the AM released from CAM in the intestine. Furthermore, the increased total-tract starch and CP digestibility were related to greater intestinal AM and trypsin activity with CAM addition. The results further showed that increasing starch digestion had a stimulatory effect on trypsin activity and CP digestion in the intestine, as reported in broilers by Jiang et al. (15). The greater CP digestibility contributed to an improvement in protein utilization efficiency, as reflected by the observed greater blood albumin and total protein contents for CAM addition, which can be used as indicators of protein utilization efficiency (39). Rumen propionate concentration for CAM and AM addition was 21.8 and 23.6 mM, respectively; however, the blood glucose concentration was greater for bulls receiving CAM supply. The results showed that supplementation with CAM had a greater improvement in intestinal digestion and energy supply of starch. When dietary starch was digested in the small intestine and rumen, the energy utilization efficiency was 60% and 48%, respectively (40). Bulls receiving CAM addition had lower ADF apparent digestibility, and this was consistent with the results that rumen carboxymethyl cellulase and cellobiase activity and *F. succinogenes*, *R. albus*, and *R. flavefaciens* were lower after CAM supplementation than AM addition. The results suggested that the AM released from CAM in the rumen probably did not support the optimum growth of cellulolytic bacteria. Furthermore, similarly observed AM activity for CAM and AM addition further suggested that the increased AM activity due to AM supply was caused by the stimulatory effects of AM on ruminal microbial growth. Exogenous AM degraded more starch into oligosaccharides, providing more substrates for microbial growth and reproduction, thereby increasing the number of microorganisms (7).

## 5 Conclusion

The supplementation of 0.6 g/kg DM AM (AM or CAM) promoted ADG and nutrient digestibility of bulls. These positive impacts were mostly caused by the increment in ruminal microbial population and intestinal digestive enzyme activity. The AM should be supplied in the form of CAM, reflected as the greater ADG observed for bulls receiving CAM compared with those consuming AM addition.

## Data availability statement

The raw data supporting the conclusions of this article will be made available by the authors, without undue reservation.

## Ethics statement

The animal study was approved by Animal Care and Use Committee of Shanxi Agricultural University. The study was conducted in accordance with the local legislation and institutional requirements.

## Author contributions

XZ: Formal analysis, Resources, Writing – original draft. FX: Resources, Writing – original draft. KX: Formal analysis, Resources, Writing – original draft. QL: Funding acquisition, Writing – review & editing. GG: Resources, Validation, Writing – original draft. WH: Data curation, Writing – original draft. YZ: Visualization, Writing – original draft. CW: Validation, Writing – review & editing.
